# *atm* Mutation and Oxidative Stress Enhance the Pre-Cancerous Effects of UHRF1 Overexpression in Zebrafish Livers

**DOI:** 10.3390/cancers15082302

**Published:** 2023-04-14

**Authors:** Yousra Ajouaou, Elena Magnani, Bhavani Madakashira, Eleanor Jenkins, Kirsten C. Sadler

**Affiliations:** 1 Program in Biology, New York University Abu Dhabi, Abu Dhabi P.O. 129188, United Arab Emirates; 2 Center for Genomics and Systems Biology (CGSB), New York University Abu Dhabi, Abu Dhabi P.O. 129188, United Arab Emirates

**Keywords:** UHRF1, liver, ataxia-telangiectasia mutated, *atm*, Tp53, zebrafish, oxidative stress

## Abstract

**Simple Summary:**

Mutation of the ataxia-telangiectasia mutated (*atm*) gene in humans and mice renders them susceptible to tumors due to both its role as a DNA damage sensor acting in pre-malignant cells to activate Tp53 and to its role in sensing and reducing oxidative stress. The oncogene UHRF1 is overexpressed in many cancers and we previously reported that UHRF1 overexpression in zebrafish hepatocytes activates a tumor suppressive pathway dependent on Tp53, resulting in senescence and a small liver which later is bypassed resulting in liver cancer. We tested the hypothesis that Atm was involved in the precancerous small liver phenotype caused by UHRF1 overexpression by generating *atm* zebrafish mutants. We show that *atm* mutation and high ROS levels enhanced, whereas antioxidant treatment suppressed, the small liver phenotype in UHRF1 overexpressing larvae. This suggests that the pre-cancerous small liver phenotype caused by UHRF1 overexpression is due to oxidative stress, which is mitigated by Atm.

**Abstract:**

The ataxia-telangiectasia mutated (*atm*) gene is activated in response to genotoxic stress and leads to activation of the *tp53* tumor suppressor gene which induces either senescence or apoptosis as tumor suppressive mechanisms. Atm also serves non-canonical functions in the response to oxidative stress and chromatin reorganization. We previously reported that overexpression of the epigenetic regulator and oncogene Ubiquitin Like with PHD and Ring Finger Domains 1 (UHRF1) in zebrafish hepatocytes resulted in *tp53*-dependent hepatocyte senescence, a small liver and larval lethality. We investigated the role of *atm* on UHRF1-mediated phenotypes by generating zebrafish *atm* mutants. *atm*^−/−^ adults were viable but had reduction in fertility. Embryos developed normally but were protected from lethality caused by etoposide or H_2_O_2_ exposure and failed to fully upregulate Tp53 targets or oxidative stress response genes in response to these treatments. In contrast to the finding that Tp53 prevents the small liver phenotype caused by UHRF1 overexpression, *atm* mutation and exposure to H_2_O_2_ further reduced the liver size in UHRF1 overexpressing larvae whereas treatment with the antioxidant N-acetyl cysteine suppressed this phenotype. We conclude that UHRF1 overexpression in hepatocytes causes oxidative stress, and that loss of *atm* further enhances this, triggering elimination of these precancerous cells, leading to a small liver.

## 1. Introduction

Senescence is a major tumor suppression mechanism that restricts proliferation of irreparably damaged or exhausted cells. A wide range of cancer-causing stimuli trigger senescence, including replicative exhaustion, DNA damage, oxidative stress, replicative stress, oncogene overexpression, NOTCH signaling and genome-wide repatterning of the epigenome [1,2]. Several tumor suppressors are implicated in senescence induction, with *tp53*-mediated cell cycle withdrawal as a hallmark of senescence in response to many, but not all cancer-causing signals [3,4,5,6]. Whether all oncogenic stimuli ultimately converge on Tp53 or whether other tumor suppressors act in parallel pathways to induce senescence or apoptosis as a mechanism of tumor suppression has not been fully elucidated.

DNA damage is assumed to be the most likely culprit of Tp53 activation in response to senescence-inducing stimuli [7,8,9]. Double and single strand breaks can activate the canonical DNA-damage sensors ataxia-telangiectasia mutated (ATM) and ataxia telangiectasia and Rad3-related (ATR), respectively. *ATM* encodes a large protein that has a kinase domain which is required for activation of the canonical DNA Damage Repair (DDR) pathway. ATM accumulates on sites of double-stranded breaks where it becomes activated and then phosphorylates hundreds of targets, including Tp53 [10]. It is well established that cell cycle withdrawal and senescence in response to ATM-mediated DNA damage sensing depends on Tp53 [7,8,9,11,12]. Since damaged cells can escape or bypass senescence by downregulating ATM or Tp53 [7,12], it is important to understand both the mechanisms by which Atm is regulated and to clarify whether Tp53 dependent or independent mechanisms account for its tumor suppressive function in vivo.

In addition to the canonical roles of ATM in the DDR, it also serves important non-canonical, kinase-independent functions, including responding to oxidative stress, regulating mitochondrial function, autophagy and apoptosis [13,14]. Homozygous *ATM* mutation underlies a human genetic disorder characterized by immune dysfunction, reproductive, mobility and neurological defects and, importantly, predisposition to cancer. These phenotypes are partially replicated in mouse and zebrafish models with *Atm* deficiency [15,16,17] and the non-cancer related phenotypes in these patients and experimental models have been attributed to functions of ATM that are unrelated to the DDR. This is supported by findings from patients with ATM mutations that affect kinase activity but leave other functions intact, and from experimental studies with ATM kinase inhibitors and kinase-dead mutants which show that these perturbations of ATM function are more potent in carcinogenesis than are null mutations [13]. These suggest that the non-canonical roles of ATM play critical functions in tumor suppression.

Modulating the response to oxidative stress and immune regulation are the best studied non-canonical functions of ATM [13,14,18,19]. ATM serves as a reactive oxygen species (ROS) sensor. ATM patients and mice with ATM mutation have increased levels of reactive oxygen species (ROS) [18,20,21,22] indicating that ATM serves to reduce ROS levels. Activation of ATM function in canonical and non-canonical pathways is dictated by differential post-translational modifications and interactions with diverse binding partners [23]. For instance, double-stranded breaks lead to ATM auto-phosphorylation, acetylation and interaction with its partner, MRN complex, to trigger a signaling pathway leading to TP53 activation. ROS accumulation also induces ATM, but through a different mechanism involving dimerization without phosphorylation. When ATM is activated in response to ROS, it promotes clearance of protein aggregates, reduction in ROS levels and maintenance of mitochondrial function [22,24,25,26,27]. This is relevant to senescent cells where ROS can accumulate. In this scenario, ATM activation could act to protect senescent cells from further oxidative damage by fortifying cellular antioxidants and dampening ROS production [18,22,28,29,30]. Interestingly, while the ROS-mediated ATM functions are Tp53 independent, oxidative stress can also act in a parallel fashion to directly modify Tp53 function [31,32,33]. Under oxidizing conditions, cysteines in the DNA-binding domain of Tp53 become oxidized [31], and since these residues are key to stabilization and function of both wild-type and mutant Tp53 [34], it is proposed that oxidative stress can activate Tp53. Moreover, Tp53 can directly impact redox balance, as loss of Tp53 can cause oxidative stress and Tp53 regulates the expression of antioxidant genes [32]. Therefore, there is a direct relationship between ATM, Tp53, oxidative stress and tumor suppression, and how this relationship functions in senescent cells in vivo has not been fully explored.

Zebrafish are a widely used cancer model and the role of Tp53 and upstream signaling pathways in cancer related phenotypes have been studied widely in this model [35,36,37,38,39]. In one study, *atm* deficiency was shown to sensitize zebrafish embryos to ionizing radiation [40] and another showed that loss-of-function *atm* mutants results in germ cell and motility defects and increases the incidence of cancer in adult zebrafish [17], mimicking phenotypes displayed by mouse mutants [15,41] and some ATM patients. Other insight came from a study showing that radiosensitivity of zebrafish embryos and mammalian cells which have compromised DDR response due to a combination of *tp53* mutation and Chk1 inhibition can be reversed by knocking down Atm [39]. A recent study using zebrafish demonstrated that loss of *atm*, but not *tp53*, protected the developmental defects caused by mutation of the telomere protecting protein Trf2 [42]. These studies indicate that Atm functions in zebrafish, as in mammals, in both Tp53-dependent and independent pathways, however, this has not yet been studied in the context of a zebrafish cancer model.

We previously reported [38] that transgenic overexpression of the human homolog of the ubiquitin-like, containing PHD and RING finger domains 1 (UHRF1) in zebrafish hepatocytes (*Tg (fabp10a:hsa.UHRF1-EGFP^mss1^)*); hereafter referred to as hUHRF1) resulted in senescence as early as 5 days post fertilization (dpf) [38]. This results in a small liver of hUHRF1 transgenics and nearly all larvae with the smallest livers die by 20 dpf, whereas those that do survive have a high incidence of hepatocellular carcinoma (HCC). The small liver phenotype and lethality is suppressed by removing 1 copy of *tp53* [38], indicating an essential role for Tp53 in precancer responses to UHRF1 overexpression. A recent study suggests that this model leads to activation of liver progenitor cells to repopulate the liver [43]. This suggests that hUHRF1 overexpression in hepatocytes activates Tp53 which induces senescence and restricts liver size, but that cancer develops in those cases where cells escape senescence or when the liver is repopulated by hepatocytes generated by liver progenitor cells [43]. In vitro studies provided a mechanistic link between ATM, UHRF1 and its partner, DNA methyltransferase 1 (DNMT1), showing that these proteins interact to regulate DNMT1 stability [44].

Here, we tested the hypothesis that *atm* functions to mediate the response to hUHRF1 overexpression in hepatocytes by generating zebrafish with loss of function mutation in *atm*. We found that, as expected, *atm*^−/−^ mutant adults were viable and that the embryos developed normally, but they were resistant to etoposide and H_2_O_2_ induced lethality and did not fully activate Tp53 or oxidative stress response genes when treated with these toxicants, respectively. Surprisingly, *atm* mutation enhanced the small liver phenotype and lethality of zebrafish with hepatocyte-specific hUHRF1 overexpression. H_2_O_2_ treatment of *Tg (fabp10a:hsa.UHRF1-EGFP^mss1^)* larvae also caused the liver to become smaller, whereas treatment with the antioxidant, N-acetylcysteine, partially rescued the small liver caused by UHRF1 overexpression. This suggests a model whereby UHRF1 overexpression in hepatocytes generates ROS and Atm activation, and that oxidative stress is an important driver of the small liver pre-cancerous phenotype caused by UHRF1.

## 2. Materials and Methods

### 2.1. Zebrafish Husbandry, Genotyping and Exposure

Adult fish were raised on a 14:10 h light: dark cycle at 28 °C. *Tg (fabp10a:hsa.UHRF1-EGFP^mss1^)* [38], hereafter called hUHRF1, were crossed to wild type (WT) zebrafish adults to generate hUHRF1^+/−^ zebrafish embryos. *tp53*^−/−^ and *atm*^−/−^ mutants [36] zebrafish were raised as incross and genotyped by PCR to identify homozygous *tp53*^−/−^ and genotyped by PCR amplification of the mutated locus followed by Sanger sequencing of the PCR product to identify homozygous *atm*^−/−^ mutants (Supplemental Table S1). *atm*^−/−^; *Tg (fabp10a:hUHRF1-EGFP^mss1^)* here after called *atm^−/−^; hUHRF1*, adults were generated by crossing *Tg (fabp10a:hUHRF1-EGFP^mss1+/−^)* to *atm*^−/−^ adults then outcrossing to *atm*^−/−^ adults and genotyping by Sanger sequencing to identify *atm^−/−^;hUHRF1* adults. These were then outcrossed to *atm*^−/−^ adults to generate embryos used for experiments. In all cases, animals were heterozygous for the hUHRF1-EGFP transgene. All animal experimentation was approved by the Institutional Animal Care and Use Committee of NYUAD (#20-0006A3).

Experiments with embryos and larvae were carried out by collecting embryos from natural spawning and maintained on a 14:10 h light:dark cycle at 28 °C in embryo medium until 5 days post fertilization (dpf). In experiments that involved monitoring juvenile fish after 5 dpf, animals were raised on a 14:10 h light:dark cycle at 28 °C and regularly feed with paramecia (1 time a day) and AP100 (2 times a day) until the day of collection.

Exposure of embryos to etoposide and hydrogen peroxide (H_2_O_2_) was carried out using standardized treatment protocols [45] in which embryos were aliquoted to a 12-well plate and treated with a range of etoposide or H_2_O_2_ concentrations to identify the optimal treatment protocol for each toxicant (Supplementary Figure S1A,B). Etoposide was resuspended in DMSO and we added 4 mL of 1 mM Etoposide (Sigma-Aldrich, St. Louis, MO) to a final concentration of 1% DMSO in embryo medium or 1% DMSO in controls. After 2 days of treatment, embryos were assessed for phenotype and collected for RNA extraction. At 120 hpf, embryos were scored for phenotype severity based on the presence of cardiac edema, lordosis and microcephaly and collected for RNA extraction. Exposure to H_2_O_2_ (VWR) was carried out by aliquoting 78 hpf embryos to a 12 well plate and adding 4 mL of 0.005% of H_2_O_2_ in embryo medium (10 embryos/well). Controls were cultured in embryo medium and all samples were assessed after 48 h of exposure (between 126–128 hpf). To assess the effects of H_2_O_2_ on liver size, larvae were treated with 0.005% at 80 hpf and assessed at 120 hpf. N-acetyl Cysteine (NAC, Sigma-Aldrich, St. Louis, MO, USA) treatment was carried out by aliquoting 4 dpf embryos, (hUHRF1 transgenics and their WT siblings) to a 12 well plate and adding 4 mL of either 0, 10, 20, 40 or 100 μM of NAC in embryo medium (10 embryos/well) and were maintained in these culture conditions for 72 h of exposure until 7 dpf when they were collected, fixed and assessed for liver size.

### 2.2. Generation of atm Mutant Zebrafish

sgRNA targeting the *atm* gene (Supplemental Table S1) was produced by sgRNA IVT kit (Takara Bio), the resulting RNA was isolated using Trizol (Invitrogen) and was quantified by Qubit. The sgRNA was diluted to 50 ng/μL, mixed with an equal volume of previously diluted nls-Cas9 protein (IDT; 0.5 μL of nl-Cas9 added with 9.5 μL of 20 mM HEPES; 150 mM KCI, pH 7.5) and incubated at 37 °C for 5 min. We then injected 1 nl into 1–2 cell stage WT embryos which were then raised to 24–72 hpf. An amount of 12–16 embryos were individually collected for genomic DNA extraction by heat shock denaturation in 50 mM NaOH (95 °C for 20 min). The remaining injected siblings were raised to adulthood to be screened for mutation using the T7 endonuclease assay. For each embryo, PCR was performed on genomic DNA by using Q5 High-Fidelity Taq Polymerase (New England Biolabs, Ipswich, MA, USA) followed by T7 endonuclease I assay (New England Biolabs) to detect mutations. For T7 endonuclease I assay, 10 μL of PCR product was incubated with 0.5 μL of T7e1 enzyme (New England Biolabs) for 30 min at 37 °C. Digested and undigested fragments were run in parallel in 2% agarose gel to assess the presence of indels. Efficiency of the mutagenesis was calculated as the number of embryos that show a positive result based on T7e1 assay divided by the total number of embryos assayed for each sgRNA. When the embryos reached sexual maturity (around 3 months), individual adult (F0) zebrafish were crossed to wild type adults to generate F1 offspring and 10–15 individual F1 embryos were collected, analyzed by T7e1 assay as previously described, and DNA from putative mutants (embryos that showed a cut in T7e1 assay) was used to perform Sanger sequencing. All 5 individual F0 adults that were screened had indel mutations, but only 2 founders had an indel (both 5 bp deletion) that was predicted to be a frameshift mutation. F1 embryos carrying this allele were raised and used to generate *atm*^−/−^ mutants.

### 2.3. Sanger Sequencing

Sanger sequencing of the *atm* allele was performed on individual F1 embryos or on tail tissue from adult zebrafish by extracting DNA using heat denaturation in 50 mM NaOH (95 °C for 20 min). PCR by using Q5 High-Fidelity Taq Polymerase (New England Biolabs) amplified a 500 bp fragment spanning the site of the mutation (Supplementary Table S1). An amount of 5 μL of the PCR products were purified by ExoSAP-IT™ PCR Product Cleanup Reagent (Thermo Fisher Scientific, Waltham, MA, USA) following manufacturer’s instruction, were sequenced using Sanger Sequencing Kit (Applied Biosystem, Waltham, MA, USA) following manufacturer’s instruction and sequenced using SeqStudio Genetic Analyzer (Applied Biosystems). Results were analyzed using Synthego (https://ice.synthego.com/#/, accessed on 20 March 2023) to identify mutant alleles. *atm*^−/−^ adults were genotyped using Sanger sequencing prior to all experiments with their offspring.

### 2.4. RNA Extraction

Larvae were collected on 5 dpf for RNA extraction from either 2–10 whole larvae for qPCR or livers microdissected from 20–30 larvae for RNA-seq. Trizol (Invitrogen, Waltham, MA, USA) was used for RNA isolation following the manufacturer’s instructions with some modifications. Briefly, 10 µg of Glycoblue (Thermo Fisher Scientific) was added during precipitation with isopropanol overnight at −20 °C. The day after, samples were centrifuged for 1 h at 12,000× *g* at 4 °C and RNA was resuspended in water and quantified using Qubit.

### 2.5. cDNA Production and qPCR

RNA was retrotranscribed to cDNA using Qscript cDNA synthesis kit with random hexamers (Quanta Bio) following the manufacturer’s instructions. cDNA was diluted 1:13 and used for qPCR using Maxima^®^ SYBR green/ROX master mix (Thermo Fisher Scientific). Gene expression levels were normalized using rplp0 gene expression by using the calculations for delta-Ct as previously described [46] (Supplementary Table S1). Changes in expression between control and treated samples were calculated as fold change (Treated/control) and the log2 was derived (L2FC). Number of clutches used for each experiment are indicated on the figures.

### 2.6. RNAseq and Bioinformatic Analysis

Total RNA extraction from approximately 20–30 livers dissected from 5 dpf zebrafish larvae for each condition was performed as previously described [47]. After extraction, RNA was treated by DNAse I followed by RNA purification (RapidOut DNA Removal Kit—Thermo Fisher Scientific). RiboZero was used to eliminate ribosomal RNA and libraries were prepared according to manufacturer’s instructions (Illumina, San Diego, CA, USA) and sequenced on NextSeq550 and HiSeq2500 (Illumina). Quality of the sequences was assessed by using MultiQC v1.0 (https://multiqc.info, accessed on 27 June 2019). After adaptor removal and trimming, reads were aligned to the *D. rerio* GRCz10 reference genome using HISAT2 with default parameters [48], mapped and counted with HTSeq [49]. Differential gene expression was calculated using a generalized linear model implemented in DESeq2 in Bioconductor [50] to test differential gene expression between hUHRF1 transgenic livers and WT sibling controls. Adjusted *p*-value with a false discovery rate of <0.05 was used to determine significantly differentially expressed genes between transgenics and controls. Datasets have been deposited in GEO (GSE227735).

### 2.7. Cy3 Streptavidin (CY3-SA) Staining and Liver Size Measurement

Cy3 staining was performed as previously described [51]. Briefly, larvae were fixed overnight at 4 °C in 4% Paraformaldehyde (Thermo Scientific, Waltham, MA, USA), washed with 0.1% PBST, dehydrated through a graded series of methanol to 100% methanol and stored overnight at 4 °C. After rehydrating through a graded series of methanol, larvae were washed with PBST, incubated in blocking solution of 3% BSA in 0.1 M NaCl/PBST (Sigma-Aldrich, St. Louis, MO, USA) for 1.5 h at room temperature. Embryos were incubated with CY3-SA (Sigma-Aldrich, 1:500) diluted in 0.1% PBST at 4 °C in the dark overnight. Following 3 washes with 0.1% PBST they were imaged on a Nikon SMZ25 stereomicroscope equipped with a fluorescent attachment for GFP and RFP (Nikon, Tokyo, Japan). ImageJ 1.53 (Dresden, Germany) was used to measure left liver lobe area by manual tracing of the liver that was marked with EGFP for hUHRF1 transgenics or marked with CY3-SA for controls.

### 2.8. Statistical Analysis

Statistical analysis was performed in GraphPad Prism 8. Number of replicates for each experiment are indicated in the figure legends. Methods to evaluate the statistical significance include two-tailed Student’s *t*-test with adjustment for multiple comparisons, long-rank test for survival curve, or Chi-square for categorical variables. Tests used are indicated in figure legend. All the plots were generated in GraphPad Prism 8 and RStudio 3.3.1.

## 3. Results

### 3.1. hUHRF1 Overexpression in Zebrafish Hepatocytes Activates the tp53 Pathway at 5 dpf

**Figure 1:**
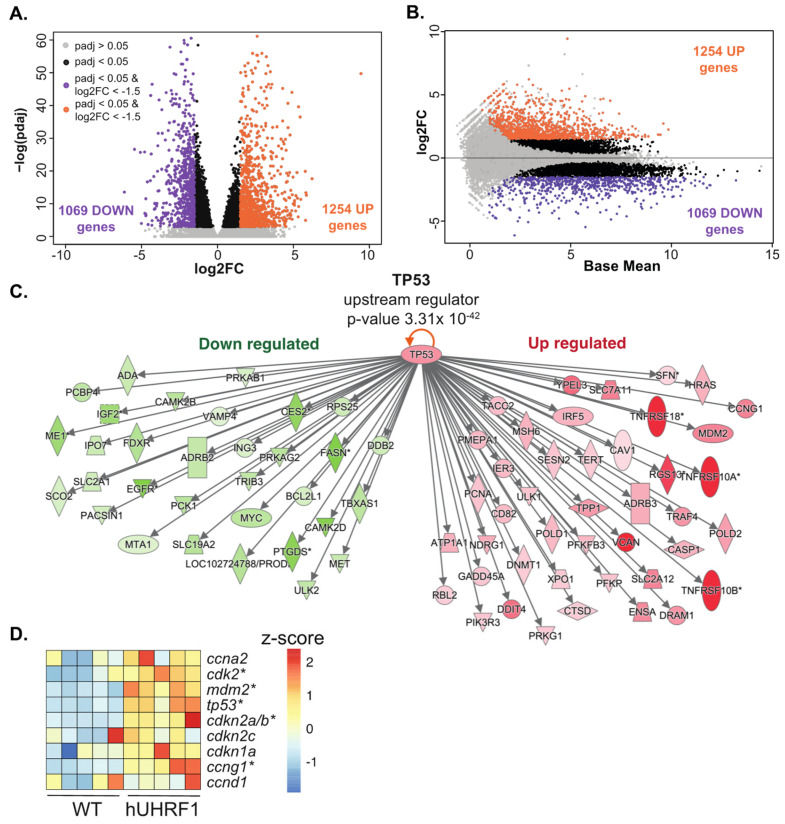
hUHRF1 overexpression in zebrafish hepatocytes activates Tp53. Bulk RNAseq analysis of 5 pools of livers collected from hUHRF1 larvae normalized to WT siblings on 5 dpf. (**A**) Volcano plot and (**B**) MA plot showing log2 fold change and log2 *p*-value adjusted of hUHRF1 overexpressing livers compared to WT siblings. Genes with *p*-value adjusted greater than 0.05 are shown in grey, significant DEGs (*p*-value adjusted smaller than 0.05) are in black, upregulated DEGs (*p*-value adjusted smaller than 0.05 and log2 fold change greater than 1.5) are in purple and downregulated DEGs (*p*-value adjusted smaller than 0.05 and log2 fold change smaller than −1.5) are in orange. (**C**) IPA analysis identified *tp53* as a top upstream regulator of the transcriptomic changes in hUHRF1 livers (*p*-value 3.31 × 10^−42^). Direct TP53 target genes predicted to be downregulated following Tp53 activation and that are downregulated in hUHRF1 overexpressing livers dataset are shown in green, and direct targets are predicted to be upregulated following TP53 activation and that are upregulated in hUHRF1 overexpressing livers. (**D**) Heatmap of the expression of TP53 direct target genes in hUHRF1 overexpressing livers compared to WT siblings at 5 dpf. Z-score is calculated on raw counts of each biological replicate. * indicates genes that have an adjusted *p*-value < 0.05.

We previously reported [38] that overexpression of hUHRF1 in zebrafish hepatocytes induced senescence as detected by increased senescence associated β-galactosidase (SA-β-gal) staining and a small liver within days of transgene expression (5–7 dpf). These transgenics had a high incidence of lethality by 15 dpf associated with a small size liver and those that did survive had a high incidence of HCC at 15 and 20 dpf. We showed that loss of one copy of *tp53* reduced SA-β-gal staining, increased liver size, reduced lethality and increased tumor incidence by 15 dpf [38]. We concluded that Tp53 activation was, at least in part, required for the hUHRF1 induced senescence and small liver phenotypes and that Tp53 acted as a tumor suppressor in this model. To better understand the downstream consequences of Tp53 activation, we performed bulk RNA-seq livers dissected from five independent clutches of hUHRF1 and six independent clutches of their WT siblings at 5 dpf, expanding our previous RNAseq analysis carried out with smaller sample sizes and lower sequencing depth [38]. The current analysis identified 5327 significant differentially expressed genes (DEGs; padj < 0.005) in hUHRF1 compared to WT at 5 dpf (Table 1). Of these, 1254 were significantly upregulated with a Log2 fold change (L2FC) of >1.5 (Table 1 and Figure 1A,B), and 1069 genes were significantly downregulated with a L2FC of <−1.5 (Table 1 and Figure 1A,B).

To identify all of the *tp53* targets and related pathways that were deregulated by hUHRF1 overexpression, we converted zebrafish genes based on Ensembl ID to human Ensembl IDs and gene names since Gene Ontology is better curated for human genes. Ingenuity Pathway Analysis (IPA) of all significant DEGs revealed liver metabolism and liver functions were the major downregulated pathways while most upregulated pathways were related to immunity and the DDR (Supplementary Table S2). Interestingly, the top upstream regulator identified by IPA was *tp53* (*p*-value 3.31 × 10^−42^, Figure 1C). Analysis of all direct TP53 targets induced or repressed by TP53 revealed that 584 genes and 18 pathways that are TP53 dependent were deregulated by hUHRF1 overexpression in 5 dpf zebrafish livers. These data validated and extended our previous findings [38] and confirms that TP53 activation is one of the primary responses to UHRF1 overexpression.

### 3.2. Deletion of atm Is Well Tolerated in Zebrafish Embryos

**Figure 2:**
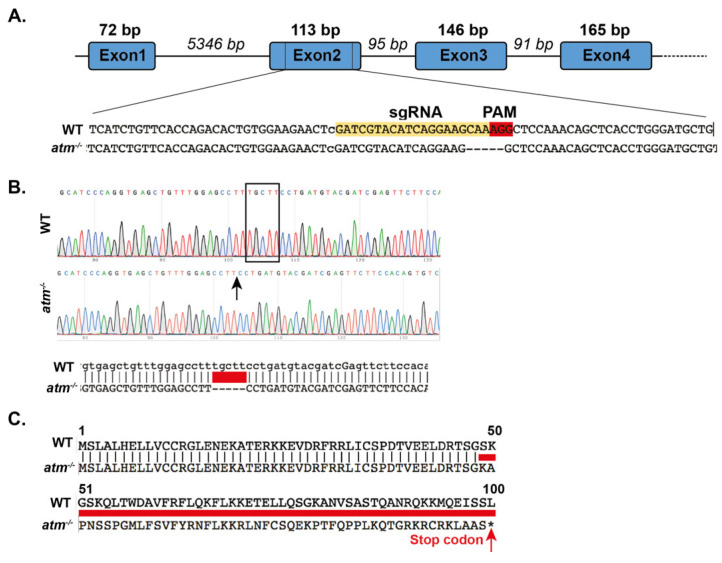
*atm*^−/−^ mutant zebrafish generated by CRISPR-Cas9. (**A**) Schematic representation of the *atm* gene from exon 1 to exon 4. Wild type and *atm*^−/−^ genomic sequences and predicted amino acid sequence of *atm* mutant alleles are shown. The sgRNA used to generate the mutant is shown in yellow, and the PAM sequence is in red. (**B**) Sanger sequencing and alignment of cDNA obtained from 2–5 dpf WT and *atm*^−/−^ mutants confirms the 5 bp deletion at the mRNA level. In red the deleted sequence. (**C**) Alignment between the predicted amino acid sequence of WT and *atm* mutant alleles. The amino acids predicted to be generated by *atm*^−/−^ mutants are indicated by the red line.

Given that ATM is a well-characterized upstream activator of TP53, and that epigenomic damage causes ATM activation in other models [9,14,52,53], we asked whether *atm* played a role in the pre-cancer phenotypes caused by UHRF1 overexpression in zebrafish. To address this, we generated an *atm* mutant line using CRISPR-Cas9 targeting of the 2nd exon of the zebrafish *atm* gene (Figure 2A). Of the five founders identified with a mutation in the target sequence, only two had a deletion that resulted in a frameshift mutation, and both had the same 5 bp deletion (Figure 2B). This deletion generates a frameshift mutation following the codon for amino acid 48, resulting in a stop codon at amino acid 100 (Figure 2C), well upstream of the kinase domain or any of the other functional features of the protein, suggesting this is a severe hypomorphic or null allele. We validated the presence of the mutation by sequencing the targeted locus in both DNA and RNA extracted from homozygous mutant embryos (Figure 2B).

**Figure 3:**
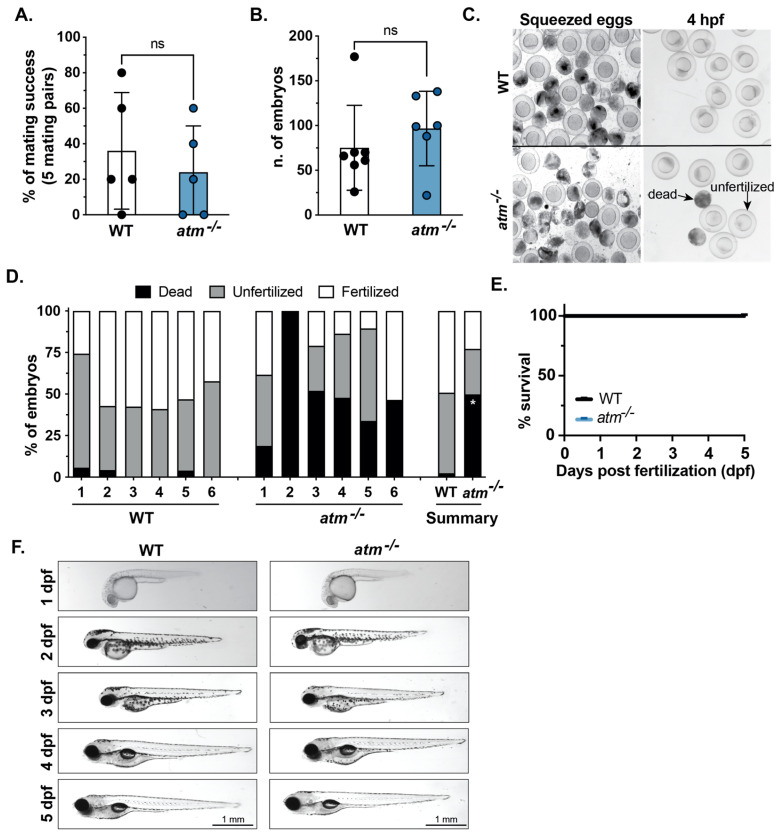
*atm* mutation is well tolerated in zebrafish embryos. (**A**) The percentage of mating success calculated from 5 individual pairs over 6 independent matings for WT and *atm*^−/−^ mutants. Each dot indicates the average success of each mating pair based on the production of any embryos. (**B**) The number of embryos generated for paired mating of WT and *atm*^−/−^ adults. Each dot indicated a different mating. (**C**) Images of WT and *atm*^−/−^ mutants after squeezing and at 4 h post-fertilization (hpf) showing fertilized, unfertilized and dead embryos. (**D**) Stack bar indicates dead, fertilized, and unfertilized as categorized in panel C for individual mating pairs of WT and *atm*^−/−^ mutants. Summary stack bars show the average of each phenotype for WT and *atm*^−/−^ mutants. (**E**) Survival curve of WT and *atm*^−/−^ mutants from 1 to 5 dpf indicates no significant differences between fertilized embryos of WT and *atm*^−/−^ mutants. (**F**) Representative images of live WT and *atm*^−/−^ mutants from 1 to 5 dpf show no observable morphological differences are detected. Values are expressed as the mean ± SD and were compared by unpaired *t*-test. Significant differences are indicated as follows: ns *p* > 0.05 (non-significant), * *p* < 0.05.

We obtained homozygous mutant (*atm*^−/−^) adults which we observed to be viable and without evidence of motility or anatomical defects up to 12 months of age (Supplementary Video S1). All adults were genotyped by sanger sequencing prior to use. Previous studies reported that *atm* zebrafish mutants were sterile [17]. We assessed fecundity and reproductive success of *atm*^−/−^ by comparing mating success of five individual mating pairs of WT and *atm*^−/−^ mated six times over a 2-month period (Figure 3A), by counting the number of embryos produced per successful mating (Figure 3B). There was no significant difference in either fecundity or number of embryos generated in each mating, indicating that *atm* mutation does not affect fecundity. However, we found variability in the number of viable eggs produced by crossing *atm*^−/−^ adults, as demonstrated by a significant increase of dead and unfertilized eggs produced in breeding of *atm*^−/−^ parents compared to WT age-matched controls (Figure 3C,D). Squeezing females to release ovary contents discharged both viable and degenerated eggs from WT animals, but mostly degenerated eggs from *atm*^−/−^ females (Figure 3C). On average, 50% and 25% of eggs from WT and *atm*^−/−^ mutants, respectively were fertilized (Figure 3D), and all of these fertilized eggs developed into normal embryos lacking any overt morphological defects through 5 dpf (Figure 3E,F). These data are consistent with findings of meiosis defects in mouse and zebrafish *atm*^−/−^ mutant gametes, but suggest that in this allele, there is a reduced penetrance of this phenotype, permitting analysis of the effects of *atm*^−/−^ on hUHRF1-overexpression mediated phenotypes.

### 3.3. atm Mutation Suppresses DNA Damage and Oxidative Stress Induced Phenotypes in Zebrafish Embryos

**Figure 4:**
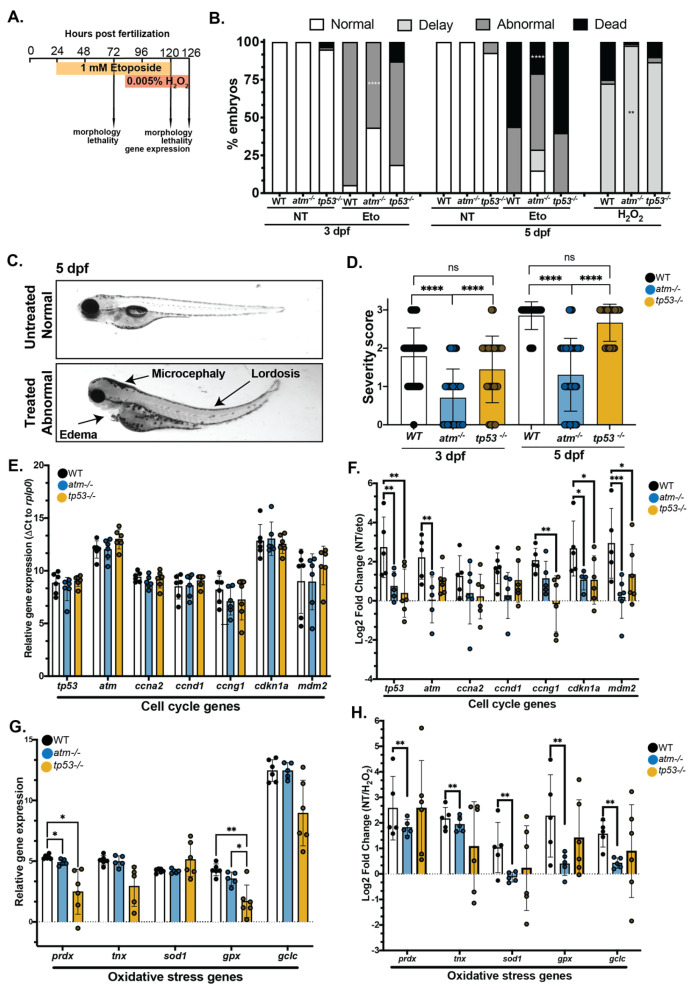
*atm* mutation suppresses the toxic response to etoposide or H_2_O_2_ exposure (**A**) Treatment scheme for exposure to etoposide and H_2_O_2_. (**B**) The percent of WT, *atm*^−/−^ and *tp53*^−/−^ embryos that have morphological defects or lethality in response to etoposide or H_2_O_2_. The experiments were performed on 6–8 clutches for each genotype and with 10 larvae per clutch (total 60 larvae). (**C**) Representative images of etoposide treated and untreated larvae at 5 dpf illustrating different phenotypes induced by etoposide treatment. Arrows indicate the phenotypes used to assign a severity score. (**D**) The phenotype severity score of etoposide treated larvae at 3 and 5 dpf in WT, *atm*^−/−^ and *tp53*^−/−^ larvae. The severity score for all untreated larvae for all genotypes was zero. The experiment was performed on 6 independent biological replicates for each genotype. Each dot represents 1 larva. (**E**) qPCR analysis of *tp53* target genes normalized to *rplp0* expression in untreated 3 dpf larvae. There are no significant differences between samples. (**F**) The log2 fold change of WT, *atm*^−/−^ and *tp53*^−/−^ 3 dpf embryos treated with etoposide compared to untreated embryos of the same genotype. The experiment was performed on 4–6 independent biological replicates for each genotype, with each dot representing values in a single clutch. (**G**) qPCR analysis of oxidative stress genes normalized to *rplp0* expression in untreated 5 dpf larvae. (**H**) The log2 fold change of WT, *atm*^−/−^ and *tp53*^−/−^ 5 dpf embryos treated with H_2_O_2_ compared to untreated embryos of the same genotype. The experiment was performed on pools of larvae from 5–6 independent clutches for each genotype, with each dot representing values in a single clutch. Values are expressed as the mean ± SD and were compared by two-way ANOVA with Tukey’s multiple comparisons test (**B**,**E**–**H**) or one-way ANOVA with Tukey’s multiple comparisons test (**D**). Only significant differences are indicated as follows: * *p* < 0.05, ** *p* < 0.01, *** *p* < 0.001, **** *p* < 0.0001.

Given the critical role of Atm in the cellular responses to double strand breaks and to oxidative stress, it is predicted that loss of Atm function would suppress the effects of these stressors. We exposed *atm* mutants to two stimuli that are well established to require Atm: etoposide, a topoisomerase inhibitor that is well-known to induce DNA double strand breaks and activate DNA damage response through Atm and H_2_O_2,_ which activates Atm via oxidative stress [18]. *tp53*^−/−^ zebrafish mutants have an impaired DNA damage response [36,39,54] and we used these as a positive control and WT embryos were used as a negative control. We optimized a treatment protocol for etoposide based on a report that treating embryos from 1–5 dpf induced DNA damage and lethality [55] and we found that during this exposure time, the lethal concentration 50 (LC_50_) was 1 mM (Supplementary Figure S1A). As H_2_O_2_ can also cause DNA damage, we aimed to expose larvae to H_2_O_2_ using a treatment protocol that generated a small amount of ROS and had only mild phenotypic effects. We confirmed that our previously established protocol in which larvae exposed from 3–5 dpf to H_2_O_2_ concentrations ranging from 0.001–0.01% represented the dynamic range for ROS production and lethality [56]. We selected 0.005% H_2_O_2_ which generated on average 26% lethality (Supplementary Figure S1B) and did not induce any Tp53 target genes in WT embryos (Supplementary Figure S2) as the optimal concentration. We used the protocol outlined Figure 4A to treat WT, *atm*^−/−^ and *tp53*^−/−^ mutants from 24–120 hpf with 1 mM Etoposide in 1% DMSO or 1% DMSO as a control and scored them for mortality and phenotypic defects at 72 and 120 hpf or with 0.005% H_2_O_2_ from 78–126 hpf. At the conclusion of the experiment, surviving larvae were collected for RNA extraction.

WT, *atm*^−/−^ and *tp53*^−/−^ embryos treated with etoposide had severe abnormalities by 3 dpf that persisted to 5 dpf (Figure 4B,C). Etoposide and H_2_O_2_ caused nearly all WT embryos to develop morphological abnormalities or premature death whereas *atm*^−/−^ mutants were protected from these effects (Figure 4B). We assessed the etoposide-induced phenotypes by assigning a severity score based on the presence or absence of 3 phenotypes: microcephaly, lordosis and edema at 5 dpf. We assigned 1 severity point for each of these phenotypes present for each treated larva for each group (Figure 4B,C), with a maximum achievable score of 3. We did not take delay into consideration since all the treated larvae showed signs of delay (no swim bladder, unconsumed yolk, underdeveloped liver and gut), consistent with the effect of etoposide in inducing DNA damage response and cell cycle arrest. *atm*^−/−^ mutants had significantly lower severity score compared to WT or t*p53*^−/−^ mutant larvae (Figure 4C). Together, these show that Atm loss reduces the cellular response to DNA damage and oxidative stress in zebrafish.

To determine if *atm* mutation had any effect on the activation of Tp53 target genes, we selected a panel of Tp53 target genes that were upregulated in hUHRF1 overexpressing livers (Figure 1D) and assessed them in 3 dpf WT, *atm*^−/−^ and *tp53*^−/−^ whole larvae. None of these genes were affected in untreated larvae (Figure 4E) and all were upregulated in etoposide-treated WT larvae as expected but were not fully upregulated in *tp53*^−/−^ and *atm*^−/−^ mutants, as expected. *tp53*, *atm*, *cdkn1a* and *mdm2* reaching statistical significance in *atm*^−/−^ mutants (Figure 4F). These data suggest that *atm* mutation partially rescues the phenotype induced by etoposide treatment and that, in absence of *atm*, Tp53 target genes cannot be fully induced in response to double stranded DNA breaks. The oxidative stress response genes *prdx* and *gpx* showed reduced expression in *atm*^−/−^ and *tp53*^−/−^ mutants in the absence of any stimuli, and upon exposure to H_2_O_2_, *atm*^−/−^ mutants failed to induce expression of all oxidative stress genes examined (Figure 4G, H). Together, these data suggest that *atm* in zebrafish larvae mediate the toxic effects of both etoposide and H_2_O_2_ exposure, independently of *tp53.*

### 3.4. atm Mutation and H_2_O_2_ Exposure Enhances the Small Liver Phenotype Caused by hUHRF1 Overexpression in Hepatocytes

**Figure 5:**
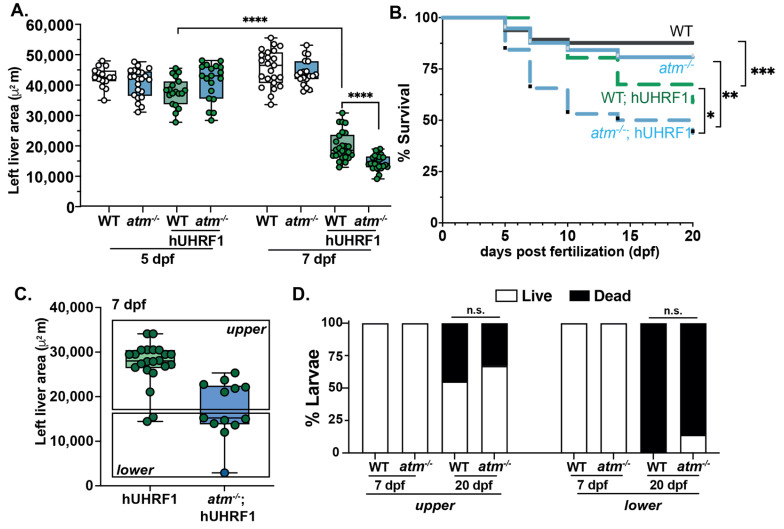
*atm* mutation enhanced the small liver phenotype and larval death in *Tg (fabp10a:hUHRF1-EGFP)* larvae. (**A**) Liver size of hUHRF1 and *atm*^−/−^; hUHRF1 larvae compared to non-transgenic WT and *atm*^−/−^ siblings at 5 and 7 dpf shows that *atm* mutation synergizes with hUHRF1 overexpression to decrease in liver size by 7 dpf. Experiments was performed in 2 biological replicates; each dot represents 1 liver. The middle line in the box plot represents the median, with the whiskers representing the range of the values for each condition. B. Survival curve of WT, *atm*^−/−^, hUHRF1 and *atm*^−/−^; hUHRF1 larvae from 5 to 20 dpf. (**C**,**D**) hUHRF1 with WT *atm* and *atm*^−/−^;hUHRF1 larvae were separated into 2 groups at 7 dpf based the liver size being above or below the median of the size measured in *atm*^−/−^; hUHRF1 larvae. Dots indicate number of livers and were assessed for survival at 7 and 20 dpf (**D**). Values are expressed as the mean ± SD and were compared by unpaired *t*-test (**A**,**C**,**D**) or by long-rank test (**B**). Significant differences are indicated as follows: n.s. indicates *p* > 0.05 (non-significant), * *p* < 0.05, ** *p* < 0.01, *** *p* < 0.001, **** *p* < 0.0001 as determined by a 2-way ANOVA.

We previously demonstrated that *tp53* heterozygosity prevented senescence and suppresses the small liver phenotype caused by hUHRF1 overexpression and accelerated HCC onset [38]. To investigate whether *atm* mutation has a similar function, we crossed *atm*^−/−^ to hUHRF1; *atm*^−/−^ adults and assessed liver size as a phenotype that is reflective of hepatocyte senescence. We assessed liver size by staining the liver with CY3-SA which binds to biotin that is enriched in hepatocytes and gut enterocytes [51]. Interestingly, while CY3-SA staining was observed in hUHRF1-overexpressing livers at 5 dpf, it was lost only in the 7 dpf hUHRF1 larvae both in presence and absence of *atm* mutation, consistent with the finding that as the larvae age, hUHRF1 overexpression reduces hepatocyte-specific factors in hUHRF1 overexpressing zebrafish (Supplementary Table S2 and ref. [43]). Therefore, to assess liver size, we measured the area of the left liver lobe using the EGFP signal in the hUHRF1 larvae and CY3-SA in non-transgenic siblings, which we found to achieve comparable results by measuring the liver area both the EGFP and CY3 signals in 5 dpf hUHRF1-overexpressing larvae (Supplementary Figure S3). We then measured the liver size of WT and hUHRF1-overexpressing larvae with and without *atm* mutation on 5 and 7 dpf. The average size of hUHRF1-overexpressing livers was smaller than WT siblings at 5 dpf, and was significantly smaller 7 dpf. Interestingly, while *atm*^−/−^ mutation alone did not affect liver size at either stage, *atm*^−/−^; hUHRF1 larvae had significantly smaller livers compared to hUHRF1 livers at 7 dpf (Figure 5A).

There was no effect of *atm*^−/−^ mutation on survival, and as expected, hUHRF1 larvae have increased lethality between 5–20 dpf which was increased significantly by in *atm*^−/−^; hUHRF1 larvae (Figure 5B). Given that liver size has been shown to correlate with survival of hUHRF1 larvae [38,43], we asked whether the increased death in *atm*^−/−^;hUHRF1 larvae was attributed to the very small livers in these animals by categorizing *atm*^−/−^;hUHRF1 and *atm^+/+^*;hUHRF1 larvae on 7 dpf based on the size of the liver as above or below the median measured in *atm*^−/−^; hUHRF1 larvae (i.e., upper/lower; Figure 5C) and tracked their survival to 20 dpf. This showed that regardless of genotype, almost no animals in the lower group survived while over 50% of larvae in the upper group survived (Figure 5D). This indicates that decreased survival in *atm*^−/−^; hUHRF1 animals is attributed to the small liver, rather than a direct effect of *atm*.

**Figure 6:**
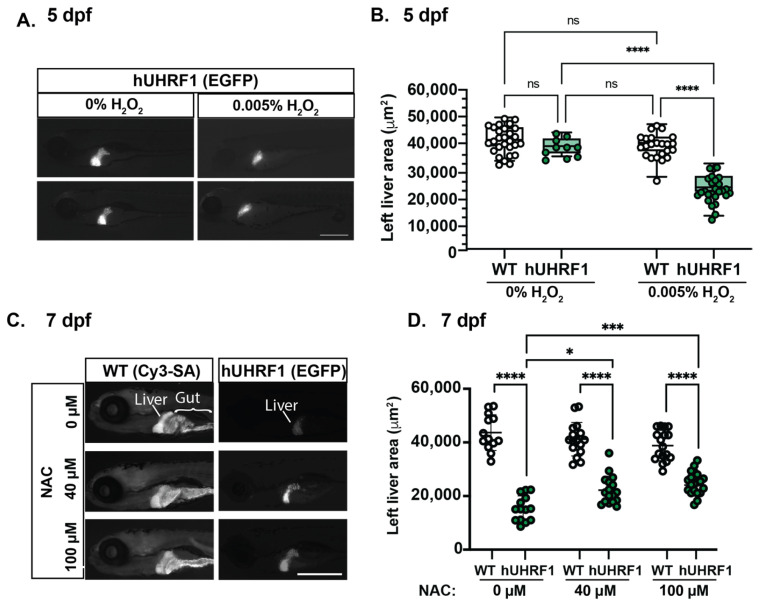
The small liver phenotype caused by hUHRF1 overexpression is enhanced by H_2_O_2_ exposure and reversed by NAC treatment. (**A**) hUHRF1 transgenic larvae and their non-transgenic siblings were untreated or treated with 0.005% H_2_O_2_ for 40–42 h and collected at 120 hpf. The left liver area was measured using the EGFP fluorescence (hUHRF1 transgenics) or CY3-SA staining (WT). (**B**) Left liver lobe area is significantly smaller in hUHRF1 larvae treated with 0.005% H_2_O_2_ compared to non-transgenic siblings treated with H_2_O_2_ and compared to untreated hUHRF1 larvae. The median is indicated by the horizontal line in the box and the whiskers represent the range of measurements. (**C**) hUHRF1 transgenic larvae and their non-transgenic siblings were untreated or treated with 40 or 100 μM of NAC from 4 dpf to 7 dpf and imaged at 7 dpf for left liver area on EGFP fluorescence (transgenics) or CY3-SA staining (controls). (**D**) Liver size is significantly smaller in hUHRF1 larvae compared to non-transgenic siblings at 7 dpf and NAC treatment increases the size of the liver in transgenic larvae. Experiments were performed in 2–3 clutches with at least 9 animals per clutch. Each dot represents 1 liver. Scale bar: 500 µm. Values are expressed as the mean ± SD and were compared by two-way ANOVA with Tukey’s multiple comparisons test (**B**,**D**). Significant differences are indicated as follows: ns *p* > 0.05 (non-significant), * *p* < 0.05, *** *p* < 0.001, **** *p* < 0.0001.

These data show that *atm* mutation enhances the small liver phenotype caused by hUHRF1 overexpression, which is the opposite of what we previously reported is the effect of *tp53* heterozygosity [38]. We therefore hypothesized that this effect could be attributed to the role of Atm in suppressing ROS [10,18,21,22,24]. If the small liver phenotype in hUHRF1 larvae is attributed to elevated oxidative stress, then these larvae should be sensitized to additional oxidative stress and protected by antioxidants. We tested this by exposing hUHRF1 and WT siblings to 0.005% H_2_O_2_; this had no effect on liver size in WT larvae without hUHRF1, but significantly decreased liver size in hUHRF1 transgenics on 5 dpf (Figure 6A,B). We next used a range of concentrations of the antioxidant N-acetyl Cysteine (NAC) which were well tolerated by WT larvae (Supplementary Figure S4) to test if this had an effect on the significant reduction in liver size in hUHRF1 larvae that we observed at 7 dpf. Strikingly, the liver in hUHRF1 larvae treated with 40 or 100 μM of NAC were significantly larger than untreated hUHRF1 larvae, although this did not restore the size to what is observed in control, non-transgenic larvae (Figure 6C,D). This suggests that the redox balance in hepatocytes is disrupted by hUHRF1 overexpression, and that oxidative stress contributes to the pre-cancerous small liver phenotype in hUHRF1 larvae.

## 4. Discussion

This study uncovers a novel role of *atm* in the hepatocyte response to overexpression of the oncogene UHRF1. In this zebrafish model, the initial cellular response to UHRF1 overexpression is activation of tumor suppression mechanisms—i.e., senescence and cell death which restrict liver growth, resulting in a small for sized liver, hepatic insufficiency and death of animals with the smallest livers; those animals that survive past 15 dpf, HCC develops with a high incidence. [47]. Here, we show that *atm* mutation in larvae that overexpress hUHRF1 in hepatocytes further decreases liver size and survival. This is surprising because we previously reported that mutation of *tp53* has an opposite effect [38]. We thus investigated the non-canonical role of Atm in sensing and mitigating oxidative stress [14,18,57], and the data we present here suggests that this function, instead of its canonical role in the DDR, interacts with hUHRF1 overexpression, and paradoxically this role increases the survival or expansion of hUHRF1 overexpressing hepatocytes. In support of this, our data show that oxidative stress also enhances the small liver phenotype of hUHRF1 transgenics and that antioxidant treatment partially rescued this phenotype. Together, these data are consistent with a model whereby hUHRF1 overexpression causes oxidative stress which is mitigated, in part, by Atm, and that mutation of *atm* causes an elevation of ROS and potentially mitochondrial dysfunction, thereby preventing expansion of liver size, leading to hepatic insufficiency and death.

Our findings implicate non-canonical roles of Atm as a tumor suppressor and point to its important function in responding to and mitigating oxidative stress [13,14,20,21,24,25]. Indeed, patients with ATM mutation, ATM mutant mice and ATM deficient cells in culture all have elevated ROS levels [25,28,58]. This can cause DNA damage, mitochondrial dysfunction and disrupt protein function, all of which can be carcinogenic. Indeed, oxidative stress is implicated in several of the clinical manifestations of ATM deficient patients [13]. These high levels of ROS can cause irreparable damage to DNA, proteins and organelles and cause cell death, ultimately eliminating Atm deficient cells and, paradoxically, act in a tumor suppressive mechanism [59]. In support of this, we found that *atm*^−/−^ zebrafish are partially resistant to the abnormal phenotypes and lethality caused by a low level of oxidative stress. It is notable that the amount of H_2_O_2_ used in these experiments had no effect on Tp53 target genes involved in the DDR and did not change the liver size in control larvae, suggesting that this level of oxidative stress did not cause DNA damage. This is further supported by our finding that Tp53 did not suppress the morphological defects caused by this low level of H_2_O_2_.

The finding that this low level of oxidative stress synergized with hUHRF1 overexpression to reduce liver size supports our hypothesis that hUHRF1 overexpression either elevates ROS or depletes antioxidant defenses. This is relevant to the clinical use of NAC, as several studies show that it can augment tumorigenesis. One study showed that NAC treatment decreased oxidative stress and cell senescence in mouse lungs, leading to reduced emphysema but increased cancer initiation [60]. Others have shown that NAC in combination with other treatments increase cytotoxicity of anticancer drugs, promoting ROS-independent apoptosis [61]. In mouse and human HCC models, one study demonstrated that NAC increased tumor growth, tumor cell migration and metastasis [62], whereas another showed that NAC decreased HCC formation in response to chemical carcinogens and TLR depletion [63]. Our data suggest that NAC could alleviate the accumulation of ROS and therefore reduce oxidative damage to sublethal levels, thereby allowing precancerous cells to survive. If this is the case, we would predict that NAC treatment will increase tumorigenesis and therefore, the use NAC as a potential cancer treatment should be considered with caution.

The results reported here support a model in which hUHRF1 overexpression activates both *tp53* and *atm*, but via different mechanisms. Our finding that H_2_O_2_ exposure synergistically enhances the small liver phenotype in hUHRF1 larvae indicates that UHRF1 overexpression may have high levels of ROS, which then activates Atm to prevents further accumulation of ROS. Recent studies suggest that downregulation of UHRF1 in cancer cells generates oxidative stress [64,65], and it is possible that the high levels of UHRF1 overexpression in this zebrafish model and in precancer cells exerts a dominant negative effect to suppress anti-oxidant defenses. Our findings point to Atm as a key factor that responds to UHRF1 overexpression and suggests the paradoxical function of Atm in pre-cancerous hepatocytes to sustain liver expansion, providing sufficient hepatic function to support survival. In this model, when *atm* is absent, the liver fails to expand and those animals with the smallest livers do not survive past 15 dpf, precluding the formation of tumors. Whether the *atm^−/−^;* hUHRF1 animals that maintain sufficient liver function to survive past 15 dpf develop liver cancer remains to be investigated. We predict that the dramatic reduction in liver size in *atm^−/−^;* hUHRF1 larvae between 5–7 dpf is due to cell death and, possibly, that hUHRF1-overexpressing hepatocytes are pruned by innate immune cells. Future experiments will be needed to determine the mechanism of liver size regulation in response to Atm loss and UHRF1 overexpression.

There are some additional questions raised by these studies that require further investigation. The allele reported here displays different phenotypes from an *atm* zebrafish mutant allele that was recently reported to have defects in germ cell development and movement [17]. It is possible that different genetic backgrounds or husbandry conditions could modify the penetrance of *atm* dependent phenotypes. An unanswered question is what is the mechanism of Tp53 activation by UHRF1? While our studies do not rule out a direct role of Atm in Tp53 activation, it is also possible that epigenetic changes induced by hUHRF1 overexpression could directly activate the expression of Tp53 or that single-stranded DNA breaks could lead to activation of ATR, Chk1 and Tp53. Our preliminary findings indicate that Atr inhibition in hUHRF1 larvae does not affect liver size, however, further analysis is required to determine if this branch of the DDR is required for Tp53 activation in this model. Finally, in these studies we use liver size as a proxy for senescence, which our previous studies have demonstrated is a response to acute overexpression of hUHRF1 in hepatocytes. Since senescence is a highly pleiotropic cellular response and lacks any definitive markers [2], we used liver size as a phenotype that is directly relevant and easy to measure. However, there are other processes that can influence organ size, and it will be important for future studies to examine how the molecular and cellular traits of senescence are affected by *atm* in this model.

## 5. Conclusions

There are three main conclusions from this study that are relevant to cancer. First, we generate a new animal model to study the important tumor suppressor, *atm*. This will provide a useful tool to complement studies in mice and in tissue culture that have highlighted the diverse functions of this gene. Second, we found that *atm* loss protects against both DNA double-strand breaks as well as oxidative stress in zebrafish. Third, we uncover a surprising role for *atm* and for oxidative stress in regulating the expansion of precancerous hepatocytes in the setting of hUHRF1 overexpression. This is highly relevant, as hUHRF1 is overexpressed in nearly all cancer types where it is proposed to function as an oncogene by generating genome instability [66,67]. Our findings suggest that in the absence of *atm*, UHRF1 overexpression is highly cellular toxic, leading to elimination of hepatocytes and, ultimately, hepatic insufficiency and larval death. This finding opens potential avenues to pursue for combination therapy that could eliminate UHRF1-overexpressing cells in the pre-cancer stages.

## Figures and Tables

**Table 1:** Differentially expressed genes in 5 dpf zebrafish livers with hUHRF1 overexpression in hepatocytes based on bulk RNA-seq analysis.

hUHRF1 vs. WT at 5 dpf	n. Genes
Total identified genes (base mean > 0)	21,642
Significant differentially expressed genes (DEGs; padj < 0.05)	5327
Significant upregulated genes (padj < 0.05 & Log2 fold change > 1.5)	1254
Significant downregulated genes (padj < 0.05 & Log2 fold change < −1.5)	1069

## Data Availability

All the datasets used in this paper are available as GEO datasets under the number GSE227735. Code used to generate the Figure 1 is available on https://github.com/SadlerEdepli-NYUAD (accessed on 19 March 2023).

## Supplementary Materials

The following supporting information can be downloaded at: https://www.mdpi.com/article/10.3390/cancers15082302/s1, Table S1: List of primers used in this study; Table S2: Ingenuity Pathway Analysis results; Video S1: *atm*^−/−^ adult fishes have no defect in their motility or morphology; Figure S1: Survival with different concentrations of Etoposide and H_2_O_2_; Figure S2: *tp53* target genes are not induced in 5 dpf larvae treated with H_2_O_2_. Figure S3: Comparison between Cy3 and EGFP method shows no significant differences between left lobe liver size measured by Cy3 or EGFP fluorescence. Figure S4: Phenotype of WT and *hUHRF1* larvae after NAC treatment.
