# Supplementary material for: atm Mutation and Oxidative Stress Enhance the Pre-Cancerous Effects of UHRF1 Overexpression in Zebrafish Livers
Source: Cancers (Basel). 2023 Apr 14;15(8):2302. doi: 10.3390/cancers15082302 (PMC10136906; doi:10.3390/cancers15082302)
Supplement: Supplementary file 1 [file cancers-15-02302-s001.zip › Supplementary materials_Sub2.pdf]

Primer ID	Application	Forward Sequence 5'-3'	Reverse Sequence 5'-3'	Source
tp53	qPCR	CAGGCCCATCCTCACAATCA	ACACGCACCTCAAAAGACCT	*New*
atm		GTGTGCTGCAGAGGACTTGA	ATCCCAGGTGAGCTGTTTGG	*New*
ccna2		CTCCATGTCTGTGCTGAGGG	TACACAAACTCCGCCACCTC	*New*
ccnd1		ACTTCCTTGCCAAACTGCCTA	GGAGGGCTTGCGATGAAGTT	*New*
ccng1		GCTCCGACTGCTCTTCACTT	AACTGTGCCTCAAGCCTCTC	*New*
cdkn1a		TCCTGAGGAGATCTGAAACCCT	CGAGTGAACGTAGGATCCGC	*New*
mdm2		TCTACCTCACAACAGCAGCG	GTCAGGGAAAAGCTGTCCGA	*New*
sod1		CGTCCATGCTTTTGGTGACAA	GTCTCCGACGTGTCTCACA	*New*
prdx1		ATCGCCTTCAGTGATGCTGC	TTTTGGTCCAGGCAAGATGGC	*New*
txn		GGATGATGCACAGGATGTGGC	CAGCTTGGATTGGTTAGATCCAGA	*New*
gpx1a		GTAAACCAGCGGCTTCTACG	GGCACTTTAATCATGACTGCAC	*New*
gclc		AACCGACACCCAAGATTCAGCACT	CCATCATCCTCTGGAAACACCTCC	*New*
sgRNA- atm	**Generation of sgRNAs**	ATTTAGGTGACACTATAGATCGTACATCAGGAAGCAAGTTTTAGAGCTAGAA ATAGC		*New*
Universal reverse primer		AAAGCACCGACTCGGTGCCACTTTTTCAAGTTGATAACGGACTAGCCTTATT TTAACTTGCTATT		*Irion et al., 2014*
atm	**T7e1 assay and Sanger**	CATAGATTCCTTGTGGGCCTGT	CGCCTGCAGAACGGAAGTATTA	New
tp53 (mut)	**Genotyping**	GATAGCCTAGTGCGAGCACACTCTT	AGCTGCATGGGGGGGAA	*Berghmans et al. 2005*
tp53(WT)		GATAGCCTAGTGCGAGCACACTCTT	AGCTGCATGGGGGGGAT	*Berghmans et al. 2005*

Supplemental Table S1: List of primers used in this study.

Supplemental Table S2: Ingenuity Pathway Analysis results. Excel sheet attached

Supplemental Video S1: *atm*$^{-/-}$ adult fishes have no defect in their motility or morphology. 14 months homozygous mutant (*atm*$^{-/-}$) adults have no evidence of motility or anatomical defects compared to WT.

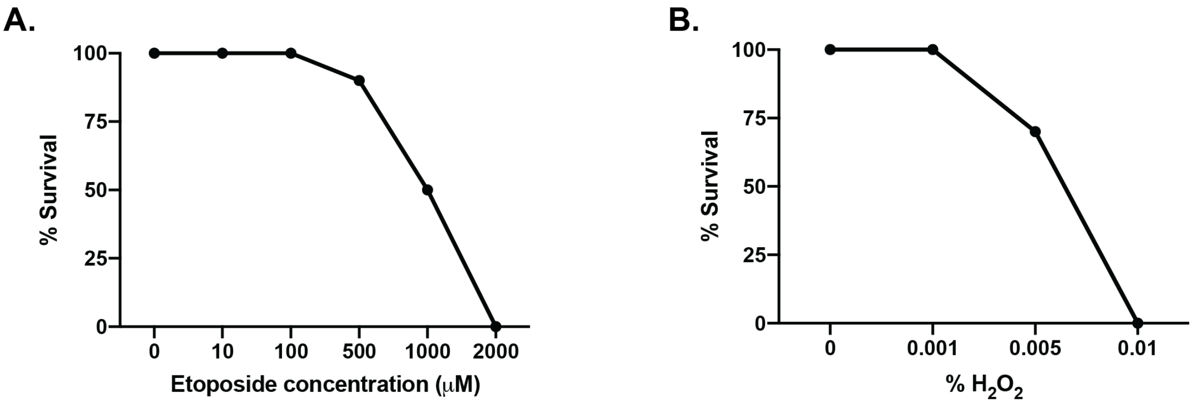

Supplemental Figure S1: Survival with different concentrations of Etoposide and H_2O_2. A. Embryos were treated with a range of etoposide or **B.** hydrogen peroxide (H_2O_2) concentrations to identify the optimal treatment protocol for each toxicant.

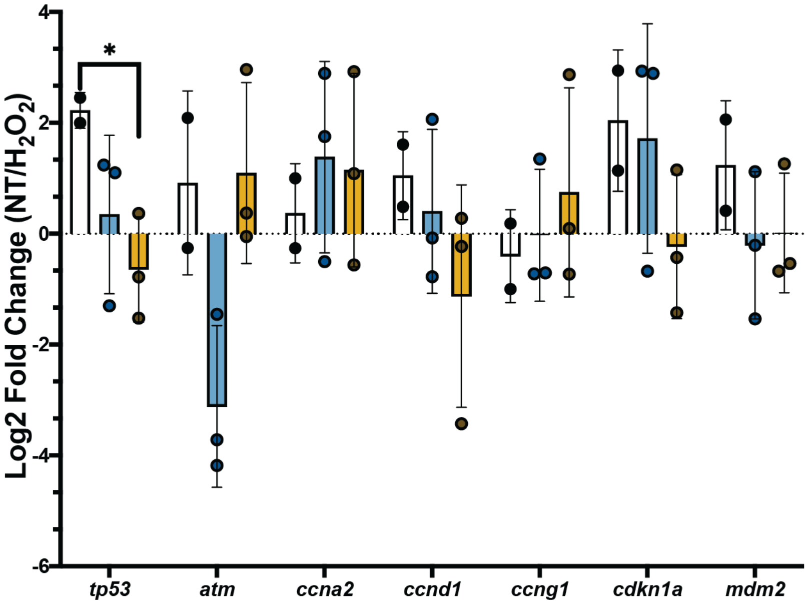

Supplemental Figure S2: *tp53* target genes are not induced in 5dpf larvae treated with H_2O_2. The log2 fold change of WT, *atm*$^{-/-}$ and *tp53*$^{-/-}$ 5dpf embryos treated with H_2O_2 compared to untreated embryos of the same genotype. The experiment was performed on pools of larvae from 2-3 independent clutches for each genotype, with each dot representing values in a single clutch. Values are expressed as the mean ± SD and were compared by two-way ANOVA with Tukey's multiple comparisons test. Only significant differences are indicated as follows: *$p<0.05$.

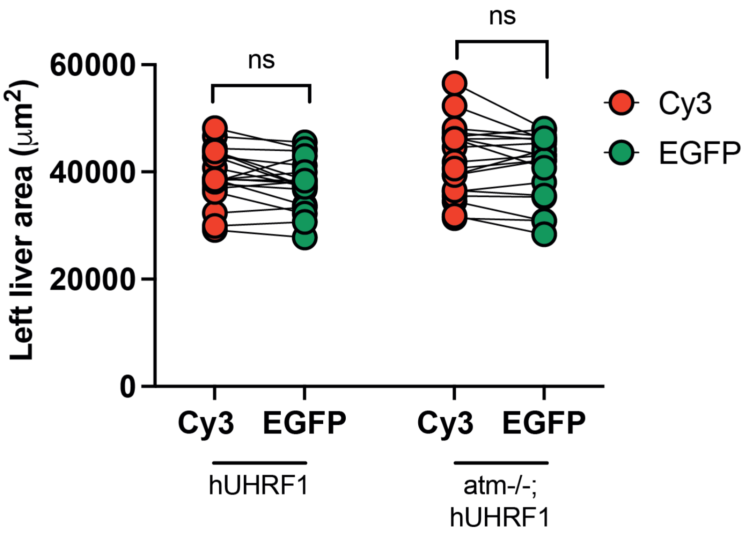

Supplemental Figure S3: Comparison between Cy3 and EGFP method shows no significant differences between left lobe liver size measured by Cy3 or EGFP fluorescence. Liver size of hUHRF1 and *atm*$^{-/-}$; hUHRF1 at 5 dpf measured by Cy3 and GFP was performed in 2 clutches with at least 6 animals per clutch. Each dot represents 1 liver. Significance was measured by unpaired t- test, ns indicates non-significant, **** indicates p-value < 0.000001.

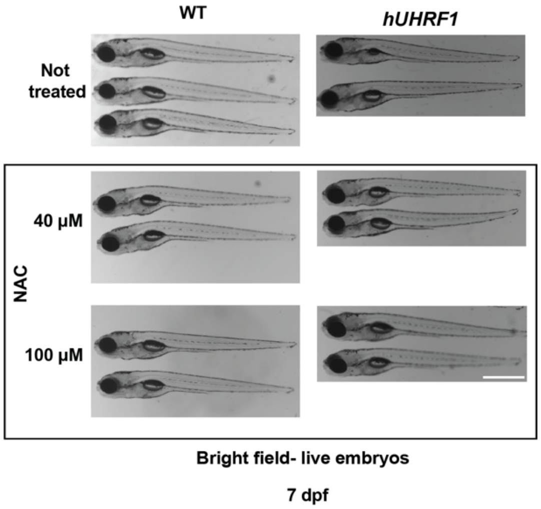

Supplemental Figure S4: Phenotype of 7dpf WT and *hUHRF1* larvae after NAC treatment. Bright field imaging of control and *hUHRF1* embryos at 7 dpf with no treatment, 40 or 100 μm of NAC show normal looking with no overt phenotype. The experiment was performed on a pool of 10 larvae from 4 independent clutches for a total of 40 embryos. Scale bar: 500 μm
